# Single-cell transcriptome profiling reveals altered neural crest cell dynamics and novel biomarkers in EDNRB mutant mice with Hirschsprung's disease phenotype

**DOI:** 10.1016/j.gendis.2025.101765

**Published:** 2025-07-08

**Authors:** Minhao Sun, Jafari Halima, Yidan Li, Jiangtian Su, Ge Yang, Jiajun Guo, Qiwen Yang, Ruihua Dang

**Affiliations:** College of Animal Science and Technology, Northwest Agriculture and Forestry University, Yangling, Shaanxi 712100, China

Hirschsprung's disease (HSCR) results from neural crest cell migration and differentiation issues.^1^ Previously, analysis was limited to bulk tissue samples, but single-cell RNA sequencing now offers microscopic insights. Our pioneering single-cell RNA sequencing analysis of colon tissue is helping unravel HSCR's origins and development. We examined 22,353 cells from wild-type (WT) and endothelin receptor type B (*EDNRB*) mutant mice, identifying 24 clusters comprising 15 cell types. Comparing HSCR-type and healthy colons revealed distinctions in cellular differentiation, transcriptome characteristics, and biological functions. Our goal is to fill knowledge gaps and uncover the molecular basis of HSCR, facilitating potential therapeutic advancements.

This study involves the use of C57BL/6J mice as the standard strain. Mice from the *EDNRB*^*m1yzcm*^ strain, a subset of N-ethyl-N-nitrosourea (ENU) mutagenic animals, exhibit HSCR characteristics in homozygous offspring. A mutation (T to C) at position 857 of the *EDNRB* gene was identified, leading to the substitution of leucine at position 286 with proline in the fifth transmembrane region. Heterozygous mice display white abdominal dots, while homozygous *EDNRB*^*m1yzcm*^ mice have a white coat on their abdomen and back, with black patches on the head and tail. Homozygous mice remain healthy in the first week after birth but typically succumb to survival limitations, often within 21 days, due to abdominal distension.

First, we analyzed 23,167 colon cells from WT mice or *EDNRB* mutant mice (experimental group, EG) (Fig. S1A). After identifying 10,817 transcriptional profiles of WT mice and 11,536 transcriptional profiles of EG mice, each cell's transcriptional profile was analyzed individually following a quality control procedure. Cells were filtered based on the number of genes reported. The t-distributed stochastic neighbor embedding (t-SNE) analysis revealed 24 subpopulations from 22,353 cells using unbiased clustering (Fig. 1A; Fig. S1C, D). Based on known conserved marker genes in each cluster, we combined clusters with comparable gene expression profiles (Fig. S1E). As a result of combining the data, 15 cell types were identified as primes (Fig. 1B; Fig. S1B). The largest subpopulations were enterocytes and enterocyte precursor cells. Although enteric neurons and enteric glial cells accounted for a small proportion, we still got some new information out of it.

**Figure 1:**
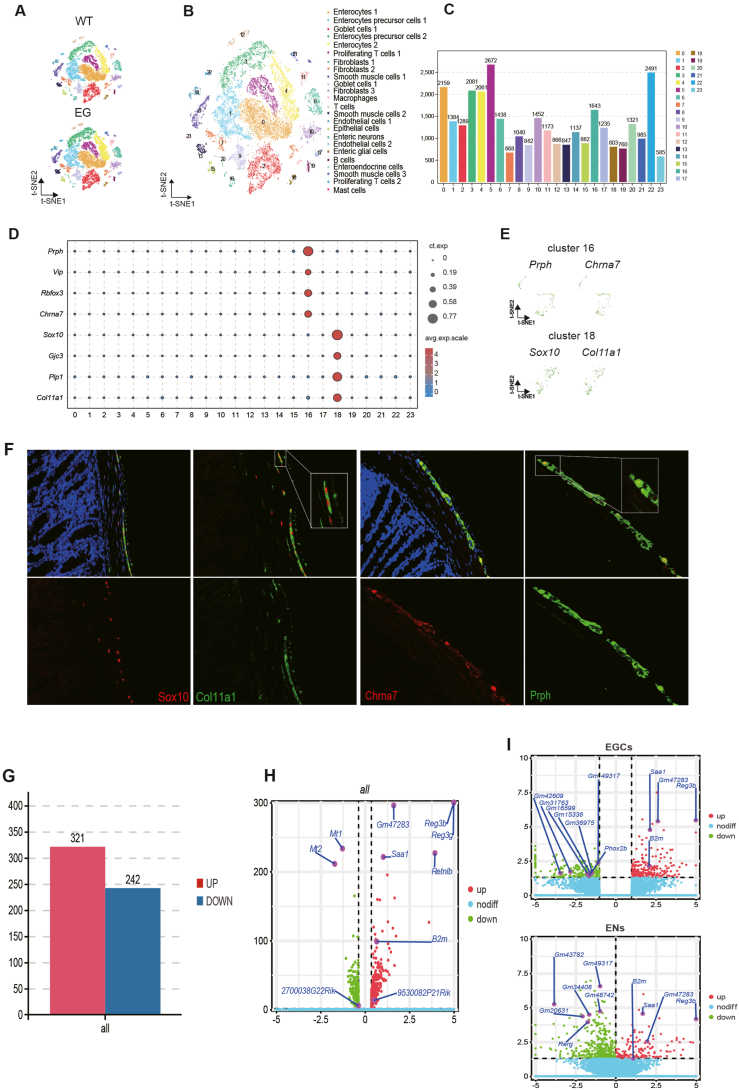
Identification of cell clusters and genes in mouse colonic tissues by single-cell RNA sequencing. **(A)** t-SNE plots of cell clusters across the indicated conditions. **(B)** t-SNE plot of per cluster. **(C)** The bar chart identifying all genes with log fold change >2 for each colonic cell type relative to all other cells. **(D)** Dot plot of some markers with strong expression in ENs and EGCs. **(E)** t-SNE plot of relative expression of specific marker genes in ENs and EGCs. **(F)** Immunofluorescence staining of tissue EN markers Prph (green), Chrna7 (red), and Prph/Chrna7, and EGC markers Sox10 (red), Col11A1 (green), and Sox10/Col11A1 overlay in colon sample. **(G)** The bar chart showing the number of up-regulated and down-regulated genes in all cell types. **(H)** The volcano map showing some up-regulated and down-regulated genes in all cell types. **(I)** The volcano map showing some up-regulated and down-regulated genes in ENs and EGCs. WT, wild type; HSCR, Hirschsprung's disease. Fibro, fibroblasts; SMC, smooth muscle cell; EC, endothelial cell; Mo/MΦ, monocyte/macrophage; T, T cell; DC, dendritic cell; Neural, neural cell; B, B cell; EN, enteric neuron; EGC, enteric glial cell; t-SNE, t-distributed stochastic neighbor embedding.

From single-cell RNA sequencing data, a complete set of differentially expressed markers for each cell population was identified (*P*-value <0.05 by Wilcoxon rank-sum test and |log_2_(fold-change)| > 1) (Fig. 1C). Figure S1F shows the top five marker genes for a cluster compared with other clusters. The expression data for around 2700 genes per cell led us to discover distinct types of cell-type-specific indicators. Using all genes to cluster cells makes it possible to identify cell subpopulations and heterogeneity at high resolution. The results are comparable to those obtained with just a few conventional cell-type-specific markers (Fig. 1D). These marker genes revealed that cholinergic receptor nicotinic alpha 7 subunit (Chrna7) might be a marker gene for enteric neurons (Log_2_fold-change = 9.62), and collagen type XI alpha 1 (Col11a1) might be a marker gene for enteric glial cells (Log_2_fold-change = 7.96) (Fig. 1E). An immunofluorescence assay and a cell count for representative markers were performed to validate and determine the anatomic location of these Chrna7^+^ and Col11a1^+^ cells. The second immunofluorescence analysis confirmed the presence of Chrna7 plus peripherin (Prph) and SRY-box transcription factor 10 (Sox10) plus Col11a1 double staining in the colonic endothelium, probably through enteric neurons and enteric glial cells. Specifically, Chrna7 was identified as a novel marker for enteric neurons, and Col11a1 was shown to be a new marker of enteric glial cells (Fig. 1F).

The canonical markers myomesin 1 (Myom1), RNA-binding protein with multiple splicing 2 (Rbpms2), and Kelch-like family member 23 (Klhl23), which are constitutively expressed throughout the mouse colon, were used to cluster all cells in the colon to identify three distinct subpopulations of smooth muscle cells (Fig. S2A, B). The Wilcoxon rank sum test was used to identify novel transcriptional markers for all colonic smooth muscle cells compared with other colonic cell populations. Some of these genes have been implicated in smooth muscle development, such as cardiac mesoderm enhancer-associated non-coding RNA (Carmn), Rbpms2, and A-kinase anchoring protein 6 (Akap6). Additionally, several genes associated with ion permeability of cell membranes have been previously identified in smooth muscle cells and other types of cells.^2^ We discovered transcriptional markers that distinguished each cluster to characterize these subpopulations. Unique markers can be defined by the Wilcoxon rank-sum test and Bonferroni correction for the highest differential expression (Fig. S2C). The first smooth muscle cell group displays potassium voltage-gated channel interacting protein 4 (Kcnip4), PDZ domain-containing ring finger 4 (Pdzrn4), and bone morphogenetic protein binding endothelial regulator (Bmper) genes linked to ion regulation, protein modulation, and endothelial cell influence. The second group features R-spondin 3 (Rspo3), collagen and calcium binding EGF domains 1 (Ccbe1), and Shisa homolog 3 (Shisa3) genes, potentially relevant to colorectal cancer and therapy. The third smooth muscle cell subgroup involves signal transduction genes like semaphorin 5B (Sema5b), Notch receptor 3 (Notch3), and laminin subunit gamma 3 (Lamc3). Two distinct enterocyte precursor cell clusters, identified by mitochondrial elongation factor Tu (Tufm), bisphosphate 3′-nucleotidase 1 (Bpnt1), and S100 calcium binding protein A1 (S100a1), exhibit different cellular focuses (Fig. S2D). In enterocyte precursor cell subgroup 1, several members of the ribosomal protein family are highly expressed, including ribosomal protein L4 (Rpl4), ribosomal protein S2 (Rps2), and ribosomal protein S17 (Rps17). The results of this study indicate that cell development in this subgroup has a close relationship with ribosome generation. On the other hand, intestinal progenitor cell subgroup 2 highly expressed solute carrier family 34 member 2 (Slc34a2), Slc15a1, Slc46a1, and other genes of the solute vector family members. This indicates that they closely resemble post-development material transport cells.^3^

We identified 321 up-regulated genes and 242 down-regulated genes significantly enriched in viral infection, cancer, mineral absorption, and tumor necrosis factor (TNF) signaling pathways (Fig. 1G, H; Fig. S3A) in all kinds of cells in the EG. Notably, the most obviously down-regulated genes were metallothionein-1 (Mt1) and metallothionein-2 (Mt2). Reduced MT1 and MT2 receptors during wakefulness suggest extended suffering.^4^ Cell counting revealed decreased enteric neurons and glial cells in EG colonic cells, aligning with prior research (Fig. S3B). Specific expression of mutation-related genes within cells further elucidates the varied roles of EG-expressed cells based on single-cell gene expression data. Numerous pathogenic genes were expressed at higher levels in enteric neurons and enteric glia cells. This is consistent with previous reports on the etiology of impaired migration and differentiation of neural crest cells (Fig. 1I). Moreover, we identified dysregulation of inflammatory genes, such as interleukin-7 (IL7) and IL18, and provided cell type-level analysis of major HSCR-associated differential genes, such as regenerating family member 3 beta (Reg3b) and paired-like homeobox 2B (Phox2b).^5^ There were 14 identical genes among the 370 up-regulated genes and 586 down-regulated genes (Fig. S3C), including Reg3b, serum amyloid A1 (Saa1), Gm47283, KAT8 regulatory NSL complex subunit 1-like (Kansl1l), serine/threonine kinase 16 (Stk16), terminal uridylyl transferase 4 (Tut4), RAS-like estrogen regulated growth inhibitor (Rerg), LysM domain containing 4 (Lysmd4), glypican 6 (Gpc6), Gm49317, Gm11149, growth arrest specific 7 (Gas7), homeobox B4 (Hoxb4), and potassium channel tetramerization domain containing 1 (Kctd1). Gene Ontology enrichment analysis showed that these genes were involved in cellular and metabolic processes (Fig. S3D).

Lastly, we examine the regulatory information associated with long noncoding RNAs and genes. In recent years, the ceRNA mechanism of lncRNA has aroused extensive research interest. Besides acting as ceRNAs, lncRNAs can also indirectly regulate miRNA target genes or specifically release target genes from miRNA inhibition. miRNA binding sites can be competitively bound by RNA molecules with specific sequence complementation sites (sequence complementing), reducing the miRNA's functional availability. Therefore, 41 lncRNAs (Fig. S3E) were extracted as risk factors for HSCR, of which 4 were up-regulated and 37 were down-regulated in all cells. Looking back at enteric neurons and enteric glial cells, we also found a lot of lncRNAs (Fig. 1I), so we think lncRNAs may affect disease development.

In this work, we identified a novel marker gene for enteric neurons and enteric glial cells by immunofluorescence co-localization. Further analysis showed that enteric smooth muscle cells and enteric precursor cells could be subdivided into distinct subpopulations. In addition, differential expression of HSCR genes was found between WT mice and EG mice. Therefore, our study may take HSCR research a step further.

## CRediT authorship contribution statement

**Minhao Sun:** Conceptualization, Data curation, Formal analysis, Software, Validation, Writing – original draft, Writing – review & editing. **Jafari Halima:** Software, Validation, Resources, Supervision. **Yidan Li:** Resources, Software. **Jiangtian Su:** Software, Visualization, Validation. **Ge Yang:** Visualization, Validation. **Jiajun Guo:** Visualization, Validation. **Qiwen Yang:** Software. **Ruihua Dang:** Investigation, Funding acquisition, Project administration.

## Ethic declaration

The experiment has been approved by the Northwest A&F University's Animal Policy and Welfare Committee (FAPWC-NWAFU; Agreement number: NWAFAC1007), which ensures animal welfare.

## Data availability

The datasets generated from this study were submitted to the National Genomics Data Center (NGDC) with accession PRJCA019890.

## Funding

This work was supported by the 10.13039/501100001809National Natural Science Foundation of China (No. 81770514, 81270439).

## Conflict of interests

The authors declare that they have no known competing financial interests or personal relationships that could have appeared to influence the work reported in this paper.

## Figures and Tables

**Figure S1:**
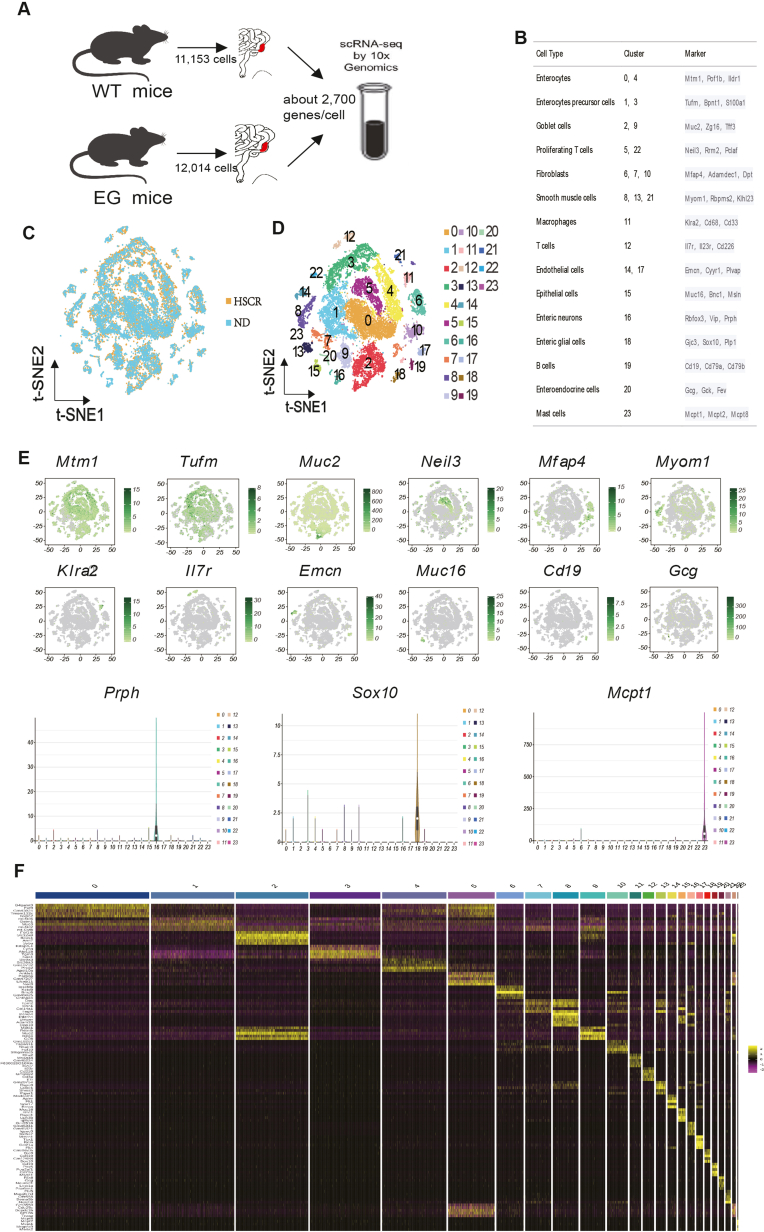
Single-cell RNA sequencing identifies novel cell markers. **(A)** Schematic diagram of the single-cell RNA sequencing program. **(B)** Major cell types and corresponding marker genes. **(C)** t-SNE plot of aggregate cells from WT and HSCR mice. After quality control, there were 10,817 cells in the WT group and 11,536 cells in the HSCR group. **(D)** t-SNE visualization of clustering, revealing 24 cell clusters. **(E)** Relative expression of several marker genes in all cells from all samples. The cells are projected onto t-SNE and violin plots. **(F)** The heatmap illustrating the top 5 markers of each colonic cell type. WT, wild type; HSCR, Hirschsprung's disease; t-SNE, t-distributed stochastic neighbor embedding.

**Figure S2:**
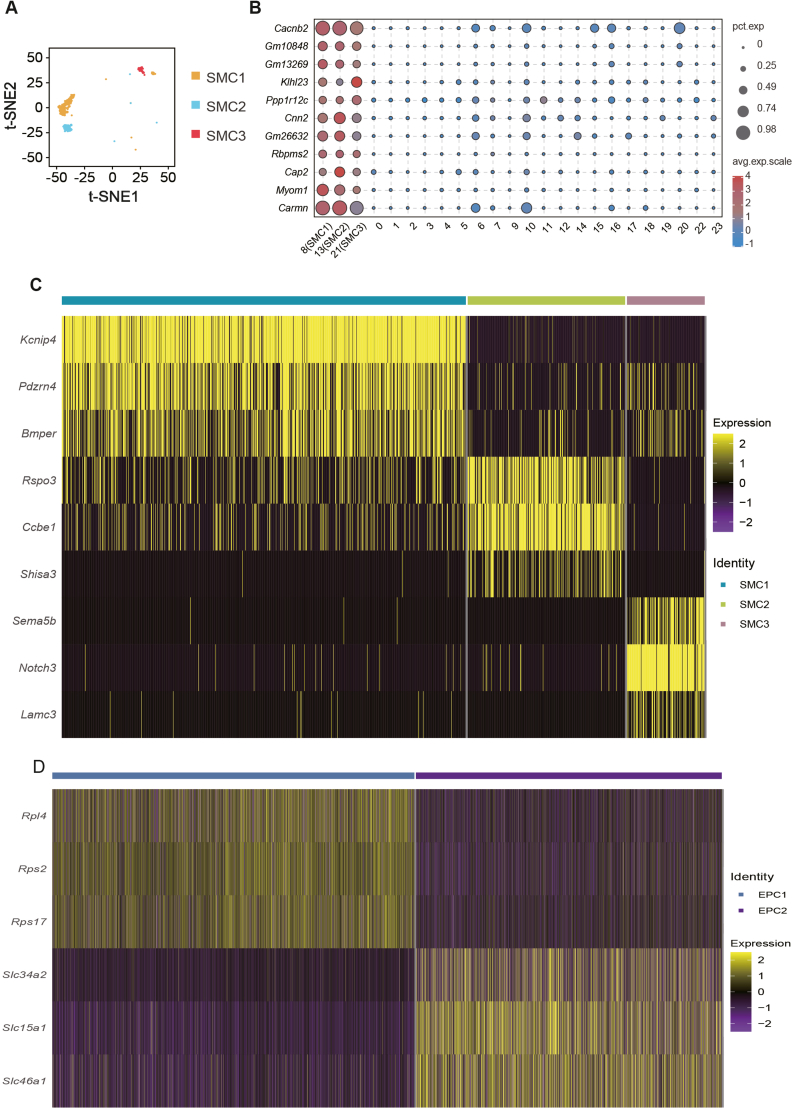
The same cell-type clusters into different distinct populations. **(A)** t-SNE plots of EC subpopulations were extracted from whole colon data and separately analyzed. In this t-SNE plot, 3 subpopulations are re-identified. **(B)** Multiple genes showed SMC-specific expression and were expressed in all 3 SMC subpopulations. A dot plot shows the percentage of cells expressing each gene (dot size) and the expression level (dot color). **(C)** Differential expression of transcription factors by SMC subpopulation. **(D)** Differential expression of transcription factors by enterocyte precursor cell subpopulation. t-SNE, t-distributed stochastic neighbor embedding; SMC, smooth muscle cell; EC, endothelial cell.

**Figure S3:**
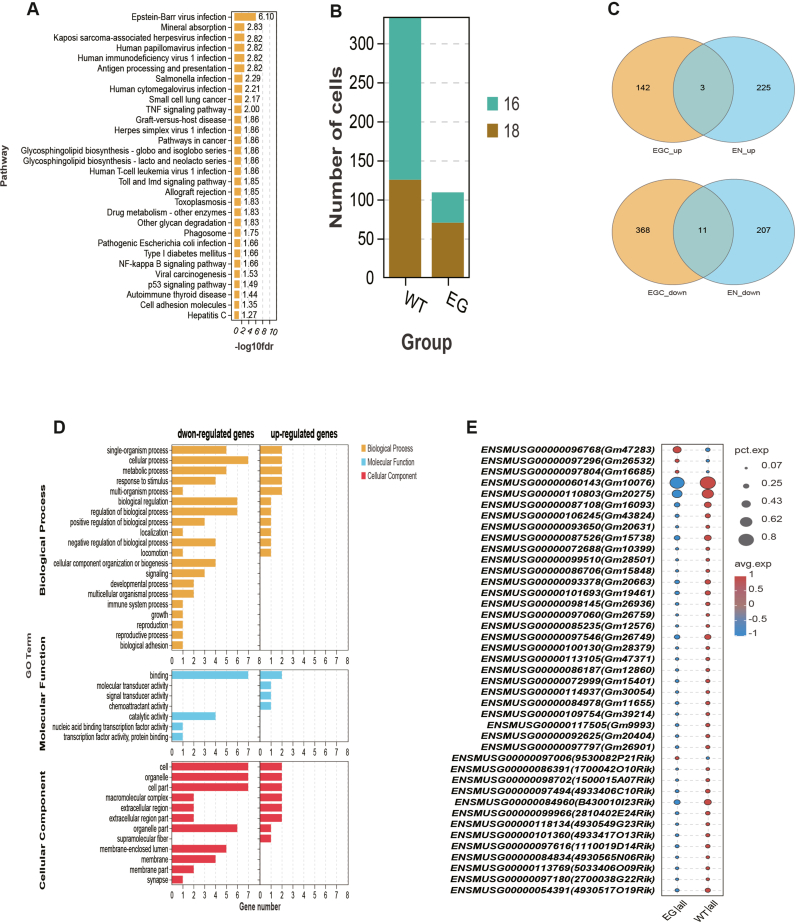
Analysis of the gene expression between HSCR and WT. **(A)** Pathway of differentially expressed genes. **(B)** Histogram of changes in the cell number of ENs and EGCs. **(C)** The same genes were screened by a Venn diagram. **(D)** Gene Ontology enrichment analysis of these same genes showed that most genes were engaged in the same CC and BP. **(E)** The bubble map shows the differential lncRNAs. WT, wild type; HSCR, Hirschsprung's disease; EN, enteric neuron; EGC, enteric glial cell; BP, biological process; CC, cellular component.
