# Supplementary material for: Single-cell transcriptome profiling reveals altered neural crest cell dynamics and novel biomarkers in EDNRB mutant mice with Hirschsprung's disease phenotype
Source: Genes Dis. 2025 Jul 8;13(1):101765. doi: 10.1016/j.gendis.2025.101765 (PMC12624628; doi:10.1016/j.gendis.2025.101765)
Supplement: Multimedia component 1 [file mmc1.docx]

**Material and Methods**

**Experimental Animals**

This study uses C57BL/6J mice as the standard strain of mice. Mice of the EDNRBm1yzcm strain were obtained from the Comparative Medical Center of Yangzhou University. This mouse model is a subset of N-ethyl-N-nitrosourea (ENU) mutagenic animals with abdominal leukoplakia. The characteristics of Hirschsprung's disease are present in homozygous mice derived from heterozygote crosses. After gene mapping and sequencing, it was found that a T to C mutation was present at position 857 of the EDNRB gene. The mutation, which occurred in the fifth transmembrane region of EDNRB, changed the leucine at position 286 to proline. White dots appear on the bellies of heterozygous mice, and homozygous EDNRBm1yzcm mice have a white coat covering their abdomen and back. Black patches appear on the head and tail. In the first week after birth, homozygous mice are healthy. Due to abdominal distension, survival is often limited to 21 days after this point. On the ninth day of the experiment, mutant and wild mice's colon tissues were collected and washed with normal saline. The samples were immediately frozen in liquid nitrogen and stored at a temperature of 80°C until needed.

**Single-Cell Capture, Library Construction, and Sequencing**

Each procedure step is summarized with the following points: (1) Cell suspensions were loaded onto a GemCode Single-Cell instrument from 10X Genomics which produces Gel Bead-In-EMlusions (GEMs) using single cells. cDNA libraries and sequencing were conducted with Chromium Next GEM Single Cell 3 Reagent Kit v3.1.(2). The post-GEM reaction mixture was cleaned with silane magnetic beads to eliminate biochemical reagents and primers. PCR was used to generate sufficient mass to construct libraries from the barcoded, full-length cDNAs. The R1 primer sequence was added during incubation in GEM. During the library’s construction, primers P5, and P7, as well as a sample index and a read 2 primer sequence were added by end repair, a-tailing, and adaptor ligation. Primers P5 and P7 are used in the final libraries for Illumina bridge amplification. (4) Single Cell 3' Libraries use standard Illumina paired-ends beginning and ending with P5 and P7. The 16 bp 10x barcode and the 10 bp UMI of Single Cell 3 were encoded in Read 1, while the cDNA fragment was sequenced in Read 2. The sample index sequences were used in the i7 index reads. A standard Illumina® sequencing primer for read 1 and read 2 was used to perform paired-end sequencing of the samples.

**Clustering Cells**

To interpret the original data, 10X Genomics Cell Ranger 3.1.0 was used. A gene matrix for each sample was obtained after filtering, quantifying, identifying, and recovering cells. The data were then imported into Seurat version 3.1.1 (Butler et al., 2018) for downstream analysis. Clustering cells involved removing those with a high number of UMIs (more than 8000) or mitochondrial genes (10%) and excluding those with less than 500 genes or more than 4000 genes. Following removing unwanted cells from the dataset, the global-scale normalization method "LogNormalize" was used to normalize gene expression measurements for each cell. The Seurat algorithm uses canonical correlation analysis and mutual nearest neighbor analysis to minimize the effects of batch effect and behavioral conditions on clustering (Schaum et al., 2020). Then, the integrated expression matrix was scaled and principal component analysis was performed to reduce the dimensions. Seurat used a graph-based clustering approach to gather the same types of cells. On the same PCs, t-distributed neighbor embedding Stochastic Neighbor Embedding (t-SNE) was generated for cluster visualization. Manually annotated cell types by consulting the literature and the CellMarker database (<http://bio-bigdata.hrbmu.edu.cn/CellMarker/>).

**Differentially Expressed Genes Analysis**

The Wilcoxon rank sum test was used to compare the expression values of genes within each cluster with those within other clusters. To identify genes with significant upregulation, a variety of criteria were used. Firstly, to qualify for the target cluster, a gene must exhibit an overexpression of at least onefold (log2(FC)>1). Second, the gene expression level in the target cluster must exceed 25%. Finally, the p-value less than 0.05 is considered significant.

**Immunofluorescence**

The sections were washed with an environmental dewaxing solution I and environmental dewaxing solution II for 10 minutes each, followed by five minutes of anhydrous ethanol I five minutes of anhydrous ethanol II, and five minutes of distilled water. After placing the slides in PBS (pH 7.4), they were washed three times for five minutes each. The slices were placed in a solution of 3% hydrogen peroxide, kept at room temperature, and protected from light for 25 minutes. After being placed in PBS (pH 7.4), the slides were purified by shaking three times on a decolorizing shaking bed to block endogenous peroxidase. PBS was dried for five minutes for each test, then BSA was added and left for thirty minutes. After removing the sealing liquid from the primary antibody, place it in a wet box at 4°C for overnight incubation. Each slide was washed three times in PBS (pH 7.4) for five minutes in the decolorizing shaker. After drying the slices with the corresponding secondary antibody labeled with HRP, the slices were incubated at room temperature for 50 minutes. Slides were washed thrice with PBS (pH 7.4) using a decolorizing shaker for each wash. After the slices were dried, TSA was added to the rings and incubated at room temperature without light for 10 minutes. Incubated slides were rinsed three times with TBST and decolored with shaders each for five minutes after incubating them. The tissue sections were placed in an antigen repair buffer-filled repair box and then microwaved. During the next 7 minutes, the fire was turned down to medium to low heat after 8 minutes. The slices were then incubated overnight at 4°C in a wet box with the primary antibody. Again, on a decolorizing shaker, slides were washed three times in PBS (pH 7.4). A mild drying process was followed by adding the fluorescent secondary antibody to the slices, which were then incubated for 50 minutes at room temperature away from light. The slides were washed three times in PBS (pH 7.4) following incubation in a decolorizing shaker. Once the slices were dried, DAPI dye was added to each circle and incubated for ten minutes away from light at room temperature. A decolorizing shaker was used to wash the slides three times in PBS (pH 7.4) for five minutes each time. Following the drying of the sections, the autofluorescence quencher B liquid was added to the ring. After 5 minutes of incubation, it was rinsed with water for 10 minutes then the anti-fluorescence quenching tablet was sealed. An image is acquired by using the following wavelengths for excitation: DAPI excitation wavelength is 330-380nm, the emission wavelength is 420nm; the excitation wavelength of 488 is 465-495nm, and the emission wavelength is 515-555nm. CY3's excitation wavelength is 510-560 nm, and the emission wavelength is 590 nm. CY5's excitation wavelength is 608-648 nm and emission wavelength is 672-712 nm.
